# The benefits and costs of domiciliary care: a study protocol for evaluating the cost-effectiveness of domiciliary care in England

**DOI:** 10.1136/bmjopen-2026-117555

**Published:** 2026-06-01

**Authors:** Florin Vadean, Georgios Mamolis, Stacey Rand, Katerina Gousia, Hansel Teo, Sarah Birch, Anica Alvarez Nishio, Ann-Marie Towers, Stephen Allan, Robin Darton, Freddie Gregory, Claire Lambert, Will Fenton, Sarah Davison, Amanda Roberts, Olena Nizalova

**Affiliations:** 1 Care and Outcomes Research Centre, University of Kent, Canterbury, UK; 2 University of Kent, Canterbury, UK; 3 Health and Social Care Workforce Research Unit, King’s College London, London, UK; 4 Ipsos UK, London, UK; 5 Skills for Care Ltd, Leeds, UK

**Keywords:** Health economics, Health policy, Quality of Life, Surveys and Questionnaires

## Abstract

**Abstract:**

**Introduction:**

Care provided in people’s own homes (domiciliary care) is an increasingly important part of long-term care. There are various services, including home visits, live-in care and housing with care. Some people directly employ care staff, called personal assistants. Services vary in quality, price and availability, and there is currently little evidence of the value these services provide to the public purse and individuals. This study protocol presents planned research to fill this important gap.

**Methods and analysis:**

This will be a cross-sectional study based on surveys of care recipients, their unpaid carers as well as formal care providers. In the first half of 2026, we will survey 1850 people accessing domiciliary care either through a homecare agency, a housing with care scheme or by directly employing personal assistants and 400 unpaid carers, all based in England. We will conduct a cost-effectiveness analysis taking a ‘production function’ approach and use quality of life as measured by the Adult Social Care Outcomes Toolkit as the main outcome of interest.

**Ethics and dissemination:**

The study received ethical approval from the School of Social Sciences Staff Review Committee at the University of Kent on 20 May 2025 (reference 1195) and the Health Research Authority, London—Camberwell St Giles Research Ethics Committee on 28 October 2025 (reference 25/LO/0652). Implications around consent, data protection and confidentiality, risk and participant payment are discussed. In addition to academic outputs (eg, academic articles, conference presentations), we aim to coproduce news items and blogs with people with lived experience of accessing long-term care and jointly present findings at events aimed at the care sector. Moreover, we will offer participating care providers benchmarking briefs based on our findings.

**STRENGTHS AND LIMITATIONS OF THIS STUDY**

- Codesigning survey materials with experts by experience and offering different survey response options to participants (ie, online, post, telephone, face-to-face) to facilitate more inclusive research.
- Measuring care outcomes using a validated quality of life measure recommended by the National Institute for Health and Care Excellence, allowing the expression of the effectiveness of different long-term care services in terms of quality-adjusted life years.
- Recruiting people accessing homecare and housing with care from four geographic regions (covering about 30 local authorities) and direct employers of personal assistants from all of England to ensure national representation.
- Facing the risk of not achieving a sufficiently large sample, despite a comprehensive strategy to avoid a low survey response rate, which could affect the identification of statistically significant differences in cost-effectiveness.
- The instrumental variable approach to infer causal effects of care services on outcomes in observational studies, although having worked well in previous research, is known to be sensitive to the instrument specification.

## Introduction

 In most Organisation for Economic Co-operation and Development countries, the focus of long-term care (LTC) provision is shifting away from institutional settings (eg, residential and nursing care) to support people to live independent lives for longer in their own homes.^1^,^3^

Similarly, in England, the ambition expressed in the Department of Health and Social Care’s policy paper on *Adult social care priorities for local authorities: 2026 to 2027* is for people to be able to access care and support earlier and closer to home.^4^ Nonetheless, there are several challenges in achieving this change. For instance, there are issues relating to care staff wages, recruitment and retention, often linked to low fee rates, which could also affect care provider quality,^5^ with providers most affected handing back contracts to public commissioners or even going out of business.^6^ Moreover, people have different support needs, values and preferences, and not all options are suitable for (or acceptable to) everyone.^1 3 7^

The size of the domiciliary care market in England is not accurately recorded. Available statistics are for the community-based (ie, non-residential) market, which includes services like day care along with services in people’s own homes, like visiting and live-in care provided by domiciliary (or home) care agencies, housing with care (eg, extra care housing or retirement villages) and services by carers directly employed by care recipients (called ‘personal care assistants’ or ‘personal assistants’; PAs). Estimates put community-based care recipients in March 2023 in the region of 585 000, with about 450 000 supported (fully or partially publicly funded) by local authorities^8^ and about 135 000 (23%) self-funding their care.^9^ Community-based care in England is provided by about 15 000 largely independent care locations and about 100 000 PAs.^10^

This study protocol has been developed following a scoping study (NIHR203902), in response to a call by the National Institute for Health and Care Research (NIHR) for research on ‘*Understanding the economic case of domiciliary care*’ (NIHR PRP 31–02-04). The scoping study included the assessment of existing evidence and available research resources (eg, data), as well as consultation with sector stakeholders, policy makers and people with lived experience of LTC about the questions and approaches for an economic evaluation study.

An international scoping literature review on cost-effectiveness analysis of domiciliary care, undertaken by members of the study team, showed that the research evidence on the economics of domiciliary care is limited and methodologically inconsistent.^11^ While many studies included both benefits and costs in their assessment of cost-effectiveness,^12^,^19^ a number of them looked only at outcomes^20^,^23^ or only at costs.^24 25^ There were also differences in outcome measures and the costs of care considered, and only a few studies used methods that considered selection problems due to unobserved confounders.^13 14 24^ All this highlighted the importance of using statistical methods that take into account selection issues when identifying the true impact of care services on care outcomes.

The assessment of existing resources did not lead to the identification of any suitable data for the evaluation of the cost-effectiveness of care services in England. National datasets (eg, the Adult Social Care Survey and the Adult Social Care Client Level Data) do not allow linking information of care inputs to care outcomes, and provider Digital Social Care Records do not include validated care outcome measures and, due to data protection issues, are not yet made available for research. Therefore, we had to include primary data collection in the research plan.

Finally, sector stakeholders confirmed during the consultation that an assessment of the benefits and costs of care services in England is highly relevant, as: (1) it could provide care commissioners with evidence on value for money of different care services and the most net beneficial option for different groups of people with care needs (eg, by age group and level of care needs); (2) it could assist policy makers in identifying the comparative value of supporting more people (ie, extending eligibility for public LTC support) as opposed to increasing the intensity of care for people already supported and (3) it could offer people with care needs self-funding their care (and their families) greater insights into the value of different care options, so more informed decisions can be made.

### Aims and objectives

The main aim of the study is to evaluate the value for money of domiciliary care. In this respect, we will assess the comparative cost-effectiveness between:

- People with different levels of care needs;
- Modes of delivery (eg, homecare agencies vs directly employed PAs);
- Different care settings (ie, residential care and housing with care).

To capture the broader societal impact of domiciliary care, we will assess its effects on healthcare demand (eg, potentially avoidable hospitalisations) as well as on unpaid carers’ quality of life (QoL) and other outcomes (eg, health and formal employment).

**Figure 1:**
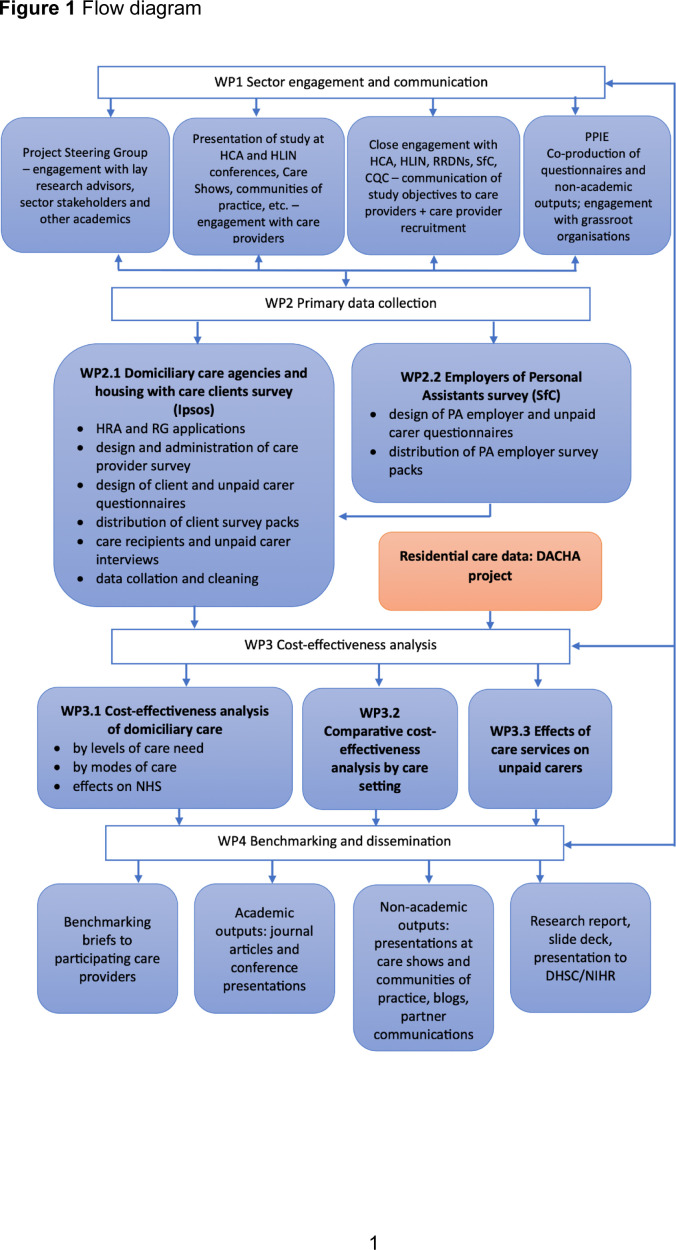
Flow diagram. CQC, Care Quality Commission; DACHA, Developing research resources And minimum data set for Care Homes’ Adoption and use; DHSC, Department of Health and Social Care; HCA, Homecare Association; HLIN, Housing Learning and Improvement Network; HRA, Health Research Authority; NHS, National Health Service; NIHR, National Institute for Health and Care Research; PAs, personal assistants; PPIE, patient and public involvement and engagement; RG, research governance; RRDN, Regional Research Delivery Network; SfC, Skills for Care; WP, work package.

To deliver our aim and objectives, we have organised the study in four work packages, as shown in the flow diagram in figure 1.

## Methods and analysis

This study will involve primary data collection of cross-sectional data that will be analysed using a ‘production function’ approach in which care outcomes (eg, social care-related QoL) are modelled as resulting from care inputs (eg, hours of care per week) and other observable factors.^13 14^

The attribution of causal effects is a challenge, as observed levels and types of care services (ie, the treatment) are dependent on both observable factors as well as factors that cannot be observed or measured; see Gousia *et al*^11^ for a review. Unobserved factors can deviate the study findings from the truth. Randomised control trials (RCTs), in which participants are randomly assigned to prespecified and controlled treatments, are considered the *gold standard* in the economic evaluation of health interventions. The adequate use of randomisation addresses this issue by balancing for observed and unobserved confounders between intervention and control groups.^26^

RCTs have been used in LTC to assess the effectiveness of smaller interventions (eg, home adaptations). For example, using the fact that people referred for the provision of an accessible showering facility by their local authority were usually put on waiting lists (control group), Whitehead and colleagues^27^ designed an RCT by randomly assigning people to receive the adaptation through a fast-tracked process (treatment group).

RCTs are, however, not always feasible or the most appropriate methodology in LTC care due to research ethics as well as internal and external validity considerations. For example, for assessing the comparative cost-effectiveness for domiciliary care by level of care need, we would have to randomly assign packages of different care intensities to people with different levels of care needs, which would be unethical. On the other hand, randomly assigning people with similar care needs to different models of care delivery would not only go against the personalisation approach in LTC in England (mandated by the Care Act 2014) but may also affect the RCT’s internal validity. People usually have strong preferences about their care, and their care arrangements can affect their QoL (eg, control over daily life is one of the domains assessed by the Adult Social Care Outcomes Toolkit (ASCOT)). Moreover, due to the complex and continuing nature of LTC, experimental settings often differ from routine practice, with substantial limitations to external validity.

An alternative approach, known as ‘instrumental variables’ (IV), has been successfully applied with observational data in LTC contexts.^13 14^ This addresses the potential issue of non-random selection (ie, we only observe the level and type of care that people have chosen or have been allocated by their local authority). We adopt the IV approach in this study. This is described in more detail in the ‘Data analysis’ section.

### Data requirements

The selected primary *care outcome* measure for people accessing care and for their unpaid carers is the ASCOT interview version (ASCOT-INT4; Netten *et al*; Malley *et al*; Malley *et al*^28^,^30^) and ASCOT-Carer INT4,^31^ respectively. These are validated instruments capturing domains of QoL that are relevant and important to adults accessing LTC services and their carers. The domains were developed based on evidence from qualitative interviews, focus groups and literature review of studies with adults accessing care and their carers to understand people’s views and perspectives on the most important areas of QoL affected by LTC.^28 31 32^ This concept/construct is known as social care-related QoL, which has been found to be sensitive to differences in satisfaction, quality and intensity of care.^33^,^36^ ASCOT has been identified as suitable and recommended for use in economic evaluation of LTC^37^ and is one of two outcome measures recommended by the National Institute for Health and Care Excellence (NICE) guidelines in LTC.^38^ An additional benefit of the use of ASCOT INT4 is that it allows the counterfactual self-estimation of the QoL effect of LTC services, as the difference between the current QoL and the ‘expected’ QoL in the hypothetical situation of absence of services.^30^

In addition to ASCOT, we will also collect data on other outcome measures. This includes potentially avoidable hospitalisations to capture the effects of homecare on avoidance of safety incidents or adverse health outcomes linked to increased demand on the NHS. For carers, we also collect data on self-reported health and employment outcomes.

Information on *care inputs* will be collected using questions on the participants’ use of paid domiciliary care (ie, hours of care per week) and other community-based care services (eg, day care, meals at home, support groups), as well as unpaid care (ie, hours of unpaid care per week).

We will also collect information on factors likely related to both care intensity and care outcomes, namely confounders. These include individual and household characteristics like age, gender, ethnicity, underlying health issues, difficulties with performing activities of daily living (ADLs), household composition, financial situation, the availability of home adaptations and general housing conditions and access to the local area and facilities. In addition, we collect information about the characteristics of the care recipients’ registered care location, namely type of service, sector and ownership; size; staffing levels; pay and employment conditions; characteristics and needs of people supported and commissioning practices of the local authorities they have contracts with, as well as the latest Care Quality Commission rating and date.

To reduce costs and avoid duplication, data on care home residents will come from the *Developing Resources and Minimum Data Set for Care Homes’ Adoption* (DACHA) study (NIHR127234). The design of survey questionnaires for this study has been informed (among others) by the structure of the data and variables collected in the DACHA project to make sure they are aligned for the comparative cost-effectiveness analysis between homecare and residential care. For example, the DACHA dataset includes information on care home residents’ QoL (collected using ASCOT Proxy), residents’ individual characteristics and care needs (eg, ADLs) and care home characteristics. The dataset includes a sample of about 679 residents from 34 care homes in England.^33^

### Recruitment of study participants

We will recruit people who access domiciliary care by using two different approaches, based on (1) whether people access care through a homecare agency or housing with a care setting or (2) whether people directly employ a PA.

For people accessing care via a homecare agency or housing with care setting, we intend to recruit 1050 participants through their registered care location. Care locations are recruited with the help of study stakeholders, including the Homecare Association, Housing LIN, NIHR Regional Research Delivery Networks, Skills for Care and the Care Quality Commission. We aim to recruit approximately 100 homecare agencies and 50 housing with care sites across England, ideally clustered in about 30 local authorities across four regions: Yorkshire and the Humber, East Midlands, West Midlands and the South East. The care provider recruitment was initiated in May 2025. We will collect key provider characteristics through a short online survey completed by managers of care locations registering to participate in the study in November 2025 to January 2026. We will distribute survey packs to their clients in February 2026. Based on previous work, we expect around 800 responses from people accessing care via a homecare agency and 250 responses from housing with care residents.

With respect to care recipients directly employing PAs, we intend to recruit 800 participants. Recruitment will take place through Mark Bates, a specialist insurance company that holds contact details of a large number of PA employers. This approach is successfully used by Skills for Care, one of our study partners, for their annual survey of PAs and PA employers. Survey packs will be sent to approximately 17 500 PA employer email addresses.

Participants who access domiciliary care will be asked to involve their unpaid carers, should they have such support. Not all participants will have unpaid care support, and some unpaid carers may not wish to participate. Based on previous studies that adopted the route of recruiting carers via adults with care and support needs, we (conservatively) anticipate collecting responses from approximately 400 unpaid carers.^39^

Survey packs to people accessing care via a care location and to people who are employing a PA will include (1) a cover letter/invitation email, (2) a participant information sheet for care recipients and one for unpaid carers, (3) the care recipient and unpaid carer questionnaires, (4) a study privacy notice and (5) two free post envelopes.

Participants will be offered the option to (1) complete a hardcopy (paper) version of the questionnaire (alone or with help), (2) complete an online version of the questionnaire (alone or with help) or (3) arrange a telephone or face-to-face interview. By providing a number of options, we are hoping to accommodate the participants’ needs and to be as inclusive as possible.

Care locations are offered financial compensation for participation in the study in accordance with the NIHR Schedule of Events Costing Attribution Tool. Also, care recipients and unpaid carers returning completed questionnaires will be offered high-street vouchers as a thank you. The value of high-street vouchers is based on NIHR guidance for payments to public contributors in research.

### Sample size calculations

Sample sizes were determined using power calculations based on differences in means of ASCOT QoL scores between groups of LTC recipients, using Adult Social Care Survey (ASCS) 2021 data. Despite its limitations (eg, it does not include people self-funding their care and does not allow the identification of homecare recipients but only people accessing a broader range of community-based services), ASCS is the largest dataset capturing outcomes of people accessing LTC in England.

For the comparative analysis between people accessing homecare and care home residents, we would expect a difference in QoL scores of <0.1 (on a scale from 0 to 1). For detecting a statistically significant difference in QoL of 0.05 and considering an SD of QoL scores of 0.189, power calculations with alpha of 0.05 and power of 80% show that we would need a minimum sample of 226 observations in each group. The comparative cost-effectiveness analysis with care home residents will need to include homecare recipients with higher levels of care needs (ie, equivalent to care needs of care home residents). Allowing for three levels of care needs for the homecare recipients group (ie, low, medium and high) and taking into account that we will not be able to control during fieldwork how many respondents will be in each care need subgroup, we rounded up the target sample of homecare recipients to 800.

For the comparative analysis between homecare agency clients and PA employers, we expect a difference in QoL of 0.02 to 0.03. For detecting a statistically significant difference in QoL and considering an SD of QoL scores of 0.188, power calculations with an alpha of 0.05 and power of 80% show that we would need a sample of between 616 and 1389 observations in each group. To avoid setting unrealistically high target samples (and increasing research costs), we aim for a similar sample of 800 PA employers, as determined in the previous step for homecare agency clients.

The very limited information on care outcomes of housing with care residents is from findings of the Adult Social Services Environments and Settings (ASSET) and the Outcomes of Social Care for Adults (OSCA) studies, which found QoL scores of around 0.90. We based the power calculation (with an alpha of 0.05 and power of 80%) on an expected difference in QoL of 0.05 and SD of 0.189, leading to a minimum sample of 226 housing with care residents, which we rounded up to 250.

### Inclusion criteria

Care recipients must be adults (ie, 18 years or over), accessing domiciliary care through a homecare agency or housing with care scheme (eg, extra care housing) or by employing a PA in England. Unpaid carers must be adult family members, partners or friends supporting a care recipient participating in the study.

All participants must have the mental capacity to consent. Our approach to mental capacity is informed by the following Mental Capacity Act principles: (1) every adult has the right to make decisions for themselves, and it must be assumed that they are able to make their own decisions unless it has been shown otherwise and (2) every adult has the right to be supported to make their own decisions. All reasonable help and support should be given to assist a person to make their own decisions and communicate those decisions before it can be assumed that they have lost capacity.

Our intention is to be as inclusive as possible in relation to people’s abilities and needs; however, we will instruct care providers to exclude people who lack the mental capacity to consent, based on their records of LTC assessment, having a court-appointed deputy or being under a Deprivation of Liberty Safeguard. We will also exclude PA employers who have an additional nominated adult (eg, a legal guardian) on their employer liability insurance.

### Consent

The way a participant’s consent will be recorded will depend on the option chosen by the participant to respond. More specifically, for the online version of the questionnaire, participants will be required to provide consent online before accessing the online questionnaire. For the hardcopy version of the questionnaire, the consent form will be included at the beginning of the questionnaire booklet, and participants will be asked to read it before completing the survey. It will be made clear that by completing and returning the hardcopy version of the questionnaire, people are consenting to participate in the study. For the telephone interview, the interviewer will offer to read out the participant information sheet and the consent form over the phone, and consent will be recorded before completing the questionnaire. Finally, for the face-to-face interview, a similar procedure will be followed, and the participant will be invited to sign a consent form.

### Data analysis

We will conduct separate cost-effectiveness analyses of domiciliary care across levels of care need (or care intensity), types of domiciliary care (eg, homecare agencies vs direct employment of PAs) and care settings (ie, domiciliary care vs housing with care vs care home services).

As mentioned earlier, we will adopt a ‘production function’ approach to estimate the cost-effectiveness of domiciliary care and use IV to address potential selection bias of care intensity with respect to unobserved factors.^13 14^

The IV method can be understood as a two-stage process. The first stage models the endogenous independent variable (ie, in our case, formal care inputs) as a function of so-called instruments and other observed characteristics. The second stage then models the outcome variable (ie, QoL) as a function of predicted values from the first stage model and the observed characteristics. Under the assumptions that the instruments are unrelated to unobserved confounders and enter the second-stage model only via the independent variable of interest (ie, affect care recipients’ QoL only through the formal care services), estimates will give the unbiased causal effect.^40^

The challenge of the IV method is to find ‘good’ instruments, namely variables (highly) correlated with care recipients’ formal care but otherwise unrelated to their QoL and other outcomes of interest. An instrument that worked well in previous studies relies on variation in local authorities’ LTC policy.^13 14^ Local authorities in England have a significant degree of autonomy in designing and commissioning LTC services tailored to population needs and local market circumstances. They also differ in the level of financial resources available to them through externally determined central government grants and council tax (ie, a tax on housing) revenue. As a result, commissioning can often differ between local authorities, and two people with the same care needs and circumstances but residing in different local areas can receive different levels of support.^13 14^ To leverage this policy variation, the above studies specified the leave-one-out average level of formal care input among individuals in a local area as the IV. This measure, also known as a spatial lag, relies on correlation between individuals’ formal care use and the level of care in their local area and is expected to be uncorrelated with unobserved individual-level confounders.

An alternative to the production function approach for identifying the impact of LTC services on care recipients’ QoL applies a counterfactual self-estimation method. ASCOT-INT4 asks participants to report both their QoL at the time of survey but also their ‘expected’ QoL in the hypothetical situation in which they would not have access to LTC services. This allows the calculation of self-reported QoL gain (ie, impact of services). Although subject to its own limitations, the methodology has been shown to be feasible and to provide similar estimates to the IV method.^30^ Therefore, where possible, we will compare these results to those of the IV analysis. Ideally, where cost-effectiveness estimates using both approaches coincide, this could provide evidence of the robustness of our findings. Where estimates differ, the direction and size of the discrepancy can also be assessed to further understand the limitations of the two approaches.

For comparative cost-effectiveness analysis between care settings, the most common approach to address the issue of self-selection bias is using matching techniques like, for example, Propensity Score Matching.^24 41 42^ By matching individuals with similar observed characteristics from the two groups (eg, gender, age, ethnicity, underlying health issues and difficulty with ADLs), the differential effect of the care services on care recipients’ QoL can be identified if assignment to groups is random conditional on controls.^43^

The same matching technique can be used for the comparative analysis between modes of domiciliary care.

### Cost-effectiveness

The estimated impact of formal care input on QoL can be seen as the marginal change in utility (ie, marginal utility $u_{k}$) arising from a marginal change in formal care input k. Care input can be measured as cost-weighted services (eg, hours of home care multiplied by gross unit costs $c_{k}$), allowing the estimation of incremental cost-effectiveness ratios, as marginal costs per marginal utility ($c_{k}/u_{k}$). Incremental cost-effectiveness ratios can be compared between different levels of care intensity, with lower values representing greater efficiency (ie, better value for money). This is relevant for informing policy questions, such as how much potential gain additional funding can achieve or how best to allocate limited budgets across different alternative services.

We also position unpaid carers as beneficiaries of care and consider the cost-effectiveness of LTC services for unpaid carers. This expands the literature on the ‘spillover effects’ of LTC services on carers to consider that services can be targeted ‘at’ or ‘for’ carers and/or jointly for supported people and their carers, based on the whole family (sometimes, known as the ‘dyadic’) approach. See the next section for more details.

Additional considerations that would generalise cost-effectiveness implications are on the interdependency between the health and LTC systems. Existing research has explored, for example, the impact of LTC spending on health service use,^44^ and our stakeholder consultation also revealed an interest in these associated effects. We will assess the effects of LTC provision on avoidable hospitalisations (ie, effects on demand for healthcare) in the analysis. However, we gave these aspects less focus, as we regard them as additional to the main aims of LTC, which is to improve the social care-related QoL of care recipients and their carers.

Differential effects between care alternatives (eg, modes of domiciliary care or care settings) will be captured by the coefficient of the (binary) ‘treatment’ variable (eg, the setting or mode of interest over an alternative) in the matching models. These coefficients will provide the average difference between the ‘treated’ groups’ actual QoL and their QoL if they would have received the alternative type of care (ie, the average treatment effect on the treated).

### Impact on unpaid carers

The analysis on the impact on unpaid carers will consider carers’ QoL and other outcomes (ie, self-reported effect of caring on employment and health) separately from those of adults accessing domiciliary care. This approach positions carers as corecipients of homecare, alongside adults with care and support needs, whose QoL and other outcomes ought to be considered in their own right.^45^ By contrast to approaches that seek to consider outcomes together (eg, Rand *et al*^34^) or determine cost-effectiveness across multiple outcomes (eg, McCaffrey *et al*^46^), we consider the cost-effectiveness of care services for care recipients and carers separately. Methodologically, it has been argued that there is a danger of ‘double counting’ or including ‘spillover effects’ that are not directly related to the intervention, especially in the evaluation of healthcare interventions.^46^

In England, however, both carer and care recipient are positioned as corecipients of LTC, with their own needs and outcomes, in policy and legislation.^45 47^ It is typically difficult to identify for each case whose services are ultimately for, due to the social and relational nature of caring.^48^ Even if a dual individual lens to cost-effectiveness analysis may overlook the relational nature of care and caring that may be more evident in qualitative or mixed methods studies,^45 49^ it allows a wider consideration of the cost-effectiveness of homecare to include the carers’ perspective, separately and in addition to that of care recipients. Regarding estimation, we will similarly apply IV estimation alongside the counterfactual self-estimation method using ASCOT-Carer INT4.^31 45^

### Patient and public involvement

We are involving six public research advisors from across England, who access domiciliary care or are unpaid carers. The advisors have been actively involved with the design of the care recipient and unpaid carer questionnaires (except for standardised tools such as ASCOT-INT4 and ASCOT-Carer INT4) and supporting materials (eg, participant information sheets) through an active and recursive coproduction process. They have provided both constructive criticism and added useful insights regarding the formulation of questionnaires (eg, length, content and format) to ensure that survey material is accessible to the target population and encourage maximum response.

The research advisors will further be involved in the interpretation of findings from statistical analysis, as well as knowledge mobilisation to non-academic audiences, for instance, grassroots organisations and communities of practice. Additionally, we recruited 10 people with lived experience of domiciliary care, either as care recipients or carers, who tested and provided feedback on the flow and content of our survey materials before we submitted them to the Health Research Authority Research Ethics Committee.

## Ethics and dissemination

The School of Social Sciences Staff Review Committee at the University of Kent approved the recruitment of registered care locations and the care location survey on 20 May 2025 (reference 1195). For the care recipients and unpaid carer survey, data analysis and knowledge dissemination, favourable ethical opinion was obtained from the Health Research Authority, London—Camberwell St Giles Research Ethics Committee on 28 October 2025 (reference 25/LO/0652).

Study findings will be published in peer-reviewed academic journals and presented at national and international conferences. The final report and other study outputs will be accessible through the project website (https://research.kent.ac.uk/dom-care/), which will include links to published peer-reviewed articles, presentations at workshops and conferences, as well as blog posts and news items. We will also coproduce non-academic outputs with our public research advisors and have joint presentations at care provider, grassroots organisation and communities of practice events.

A plain English summary of findings will be shared with participating care providers, as well as the people they support who have participated in the study. We will also offer participating care providers benchmarking briefs, illustrating their achieved care outcomes as compared with national and (where available) regional averages and distributions. These may help care providers identify, or confirm, aspects of their care services they are delivering particularly well and other aspects on which they could improve.

## Discussion

This study aims to determine the impact of domiciliary care on the QoL of people accessing care and their unpaid carers, as well as the value for money provided by domiciliary care services compared with other forms of LTC.

We will survey 1850 people accessing domiciliary care and 400 unpaid carers. A ‘production function’ approach will be used to estimate the cost-effectiveness of care services and an IV method to address potential selection bias of care intensity with respect to unobserved confounders. The study adds to previous findings by analysing data from a larger and more diverse sample of people accessing domiciliary care, people accessing different forms of domiciliary care and accounting for the role of care provider characteristics. Our contribution to the impact of domiciliary care services on unpaid carers will be through comparisons not covered in previous studies (eg, different forms of domiciliary care, types of housing, as well as sources of funding).

The main research question addressed is how much QoL gain can be attained if an additional unit of a specific type of care, for example, an hour of contact of domiciliary care, is made available? This focus on the incremental cost-effectiveness of services is relevant for informing policy questions, such as the extent to which additional funding can increase potential gains or how best to allocate limited budgets across different alternative services. This focus aligns with the needs of and feedback from policy stakeholders and the funder.

Our ambition is for our research to have an impact on several domains of the LTC system in England and the people accessing it. Project findings could inform policy decision-making to support people with care needs to live more independent lives, provide guidance about domiciliary care options, as well as support individuals and families to make more informed decisions about care. More specifically, estimates of the relative cost-effectiveness of domiciliary care could help policy makers setting public budgets (eg, inform Spending Reviews) and developing guidance to encourage home and community-based care options (eg, White Papers). Research findings could also inform practice, especially care commissioning choices by both public commissioners and individual purchasers (eg, through NICE guidance). Finally, a more direct impact on the QoL outcomes of people who access domiciliary care could be achieved through the benchmarking briefs provided to participating care providers, which may help managers identify aspects of their services where improvement would make most difference.

We also have to acknowledge a number of risks and limitations of this study. For example, the analysis relies on the successful recruitment of sufficiently large samples of homecare agency clients, housing with care residents and PA employers. Not achieving minimum sample requirements may limit the identification of statistically significant differences in cost-effectiveness, where this may exist in reality. The study team took a rather conservative approach when considering expected response rates and planned a comprehensive strategy to reduce the risk of a potential low response, including: (1) sub-contracting the data collection activity to social research specialists, (2) sending client communications and reminders through recruited providers, (3) offering alternative options of completing the survey (ie, paper, online, telephone or face-to-face), (4) setting up a helpline to respond to participants’ queries and arrange interviews (including in languages other than English), (5) visits to housing with care sites by the fieldwork team to encourage and support survey completion and (6) offering respondents high-street vouchers as a ‘thank you’ for taking part. We trust that these mitigating actions will help achieve sufficiently large and balanced subsamples.

A limitation related to methodology is that while the IV approach has been successfully used in previous observational studies to infer causal effects of care services on outcomes, it is well-known to be sensitive to the instrument specification. While our results will rely on the choice of instruments, we will test, nonetheless, for their validity and underlying assumptions. Moreover, where possible, we will also compare the results from the IV approach with the estimations of ASCOT-INT4 self-reported QoL gain (ie, impact of services). The modelling of QoL outcomes on care inputs will also rely on the choice of function form and assumptions made in this regard. The robustness of results will be tested across different functional forms.
