# Supplementary material for: The benefits and costs of domiciliary care: a study protocol for evaluating the cost-effectiveness of domiciliary care in England
Source: BMJ Open. 2026 Jun 1;16(6):e117555. doi: 10.1136/bmjopen-2026-117555 (PMC13239640; doi:10.1136/bmjopen-2026-117555)
Supplement: Supplementary file 1 [file bmjopen-2026-117555.reviewer_comments.pdf]

PEER REVIEW HISTORY

BMJ Open publishes all reviews undertaken for accepted manuscripts. Reviewers are asked to complete a checklist review form and are provided with free text boxes to elaborate on their assessment. These free text comments are reproduced below.

ARTICLE DETAILS

Title (Provisional)

The Benefits and Costs of Domiciliary Care: a study protocol for evaluating the cost-effectiveness of domiciliary care in England

Authors

Vadean, Florin; Mamolis, Georgios; Rand, Stacey; Gousia, Katerina; Teo, Hansel; Birch, Sarah; Alvarez Nishio, Anica; Towers, Ann-Marie; Allan, Stephen; Darton, Robin; Gregory, Freddie; Lambert, Claire; Fenton, Will; Davison, Sarah; Roberts, Amanda; Nizalova, Olena

---

VERSION 1 - REVIEW

**Reviewer**	**1**
**Name**	**Stirrup , Oliver**
**Affiliation**	**University College London, Institute for Global Health**
**Date**	**01-Apr-2026**
**COI**	**None**

Vadean et al. present a protocol on the study of domiciliary care in England. This is an important topic for the field of social care research, and this study has the potential to generate useful data and findings. I have some mostly minor comments and suggestions regarding the text of the manuscript, with one more substantial query. I am a statistician, with experience of research in a social care setting.

Major query: Were any calculations carried out to justify the overall and subgroup sample sizes? I know that this is not always easy, and can require simplifications and assumptions etc., but it would be good to know the basis for the choices made.

Minor points:

-I think that it would make sense to mention the use of ASCOT/QoL as the main outcome measure in the Abstract.

-Under 'Methods and analysis': "An alternative approach, successfully implemented in the LTC context with observational data, is to use instrumental variables (IV) to address selection

of care intensity on unobserved factors". Perhaps check the wording here to ensure that it can be understood more fully/easily by someone with limited knowledge of IV methodology.

-Under 'Recruitment of study participants', should "...we intend to recruit 1,850 participants through their registered care location" instead be '1,050 participants'? i.e. "800 responses from people accessing care via a homecare agency and 250 responses from housing with care residents".

-"For people directly employing a PA, we intend to recruit 800 participants. Recruitment will take place through Mark Bates Ltd, a specialist insurance company that holds contact details of a large number of PA employers." Assuming that I have understood correctly, would it make sense to specify here that the 'PA employers' are the care recipients themselves? I think that making this more explicit might help the reader, particularly if reading through quickly.

-Are there any ‘back up’ approaches to recruitment if the numbers for each group are lower than planned?

VERSION 1 - AUTHOR RESPONSE

Reviewer: 1. Dr. Oliver Stirrup , University College London

Comments to the Author:

Vadean et al. present a protocol on the study of domiciliary care in England. This is an important topic for the field of social care research, and this study has the potential to generate useful data and findings. I have some mostly minor comments and suggestions regarding the text of the manuscript, with one more substantial query. I am a statistician, with experience of research in a social care setting.

Response: Thank you very much for the very useful comments and suggestions.

Major query: Were any calculations carried out to justify the overall and subgroup sample sizes? I know that this is not always easy, and can require simplifications and assumptions etc., but it would be good to know the basis for the choices made.

Response: Thank you very much for flagging this. We carried out power calculations to determine the subgroup sample sizes for the NIHR funding application but this was omitted from the manuscript submitted. We now included this discussion under the ‘Sample size calculations’ heading on page 8.

Minor points:

-I think that it would make sense to mention the use of ASCOT/QoL as the main outcome measure in the Abstract.

Response: We included a mention to ASCOT QoL as main outcome of interest in the Abstract.

-Under 'Methods and analysis': "An alternative approach, successfully implemented in the LTC context with observational data, is to use instrumental variables (IV) to address selection of care

intensity on unobserved factors". Perhaps check the wording here to ensure that it can be understood more fully/easily by someone with limited knowledge of IV methodology.

Response: We amended the paragraph to: “An alternative approach, known as ‘instrumental variables’ (IV), has been successfully applied with observational data in LTC contexts. This addresses the potential issue of non-random selection (ie, we only observe the level and type of care that people have chosen or have been allocated by their local authority). We adopt the IV approach in this study. This is described in more detail in the ‘Data analysis’ section.” We hope this is now clearer.

-Under 'Recruitment of study participants', should "...we intend to recruit 1,850 participants through their registered care location" instead be '1,050 participants'? i.e. "800 responses from people accessing care via a homecare agency and 250 responses from housing with care residents".

Response: Thank you for spotting this typo. We have amended accordingly.

-"For people directly employing a PA, we intend to recruit 800 participants. Recruitment will take place through Mark Bates Ltd, a specialist insurance company that holds contact details of a large number of PA employers." Assuming that I have understood correctly, would it make sense to specify here that the 'PA employers' are the care recipients themselves? I think that making this more explicit might help the reader, particularly if reading through quickly.

Response: This is a good point. We have amended the wording to: “With respect to care recipients directly employing PAs,...”

-Are there any ‘back up’ approaches to recruitment if the numbers for each group are lower than planned?

Response: We do not have a ‘back up’ approach as such if recruitment numbers are lower than planned. We have added though a discussion on the risk of a potential low response to the survey and our comprehensive strategy to mitigate it in the ‘Discussion’ section.
